# Comparison of Cortical Bone Trajectory and Percutaneous Pedicle Screw Fixation in PETLIF: Short-Term Perioperative Muscle Injury and Pain Outcomes

**DOI:** 10.3390/jcm15187174

**Published:** 2026-09-15

**Authors:** Daisuke Ukeba, Ken Nagahama, Yoshinori Hyugaji, Katsuhisa Yamada, Hideki Sudo, Tsutomu Endo, Takashi Ohnishi, Ryo Fujita, Yoshinao Koike, Akira Fukushima, Norimasa Iwasaki

**Affiliations:** 1 Department of Orthopaedic Surgery, Hokkaido University Hospital, Sapporo 060-8648, Hokkaido, Japan; 2 Department of Orthopaedic Surgery, Sapporo Endoscopic Spine Surgery, Sapporo 065-0016, Hokkaido, Japan

**Keywords:** full-endoscopic spine surgery, full-endoscopic transforaminal approach lumbar interbody fusion, percutaneous endoscopic transforaminal lumbar interbody fusion, Percutaneous pedicle screw, cortical bone trajectory screw, minimally invasive surgery

## Abstract

**Background/Objectives**: Full-endoscopic transforaminal approach lumbar interbody fusion using the percutaneous endoscopic transforaminal lumbar interbody fusion (PETLIF) system is increasingly adopted as a minimally invasive technique for degenerative lumbar disorders. Screw trajectory selection may influence tissue handling, muscle injury, and postoperative pain. Comparative evidence between percutaneous pedicle screws (PPS) and cortical bone trajectory (CBT) screws in PETLIF is limited. This retrospective, non-randomized comparative study evaluated short-term outcomes between CBT and PPS fixation, focusing on muscle injury and early postoperative pain. **Methods**: This retrospective study included 140 patients who underwent single-level PETLIF between July 2021 and June 2025 (PPS: 69; CBT: 71). Allocation to PPS or CBT fixation was based on surgeon preference. Operative time, blood loss, hemoglobin (Hb) and creatine kinase (CK) levels, slip correction, Japanese Orthopaedic Association (JOA) scores, and visual analog scale (VAS) scores for low back pain were evaluated. Direct decompression was performed more frequently in the CBT group. Laboratory data were collected preoperatively and on postoperative days 1, 3, and 7. **Results**: Blood loss was higher in the CBT group due to more frequent decompression, although postoperative Hb changes were similar. CK elevation on postoperative day (POD) 1 was associated with lower values in the CBT group (753.0 ± 276.7 U/L vs. 539.3 ± 322.4 U/L). Slip correction and JOA improvement were comparable. Early postoperative low back pain (POD 7) was significantly lower in the CBT group (3.2 ± 2.2 vs. 2.3 ± 1.9). **Conclusions**: CBT fixation in PETLIF was associated with lower short-term muscle injury and early postoperative pain while maintaining radiological and functional outcomes comparable to PPS. However, the higher frequency of direct decompression in the CBT group represents an important potential confounder. This study evaluated only ultra-short-term perioperative outcomes up to 60 days; long-term endpoints such as fusion rate, cage subsidence, screw loosening, and adjacent segment disease were not assessed.

## 1. Introduction

**Figure 1:**
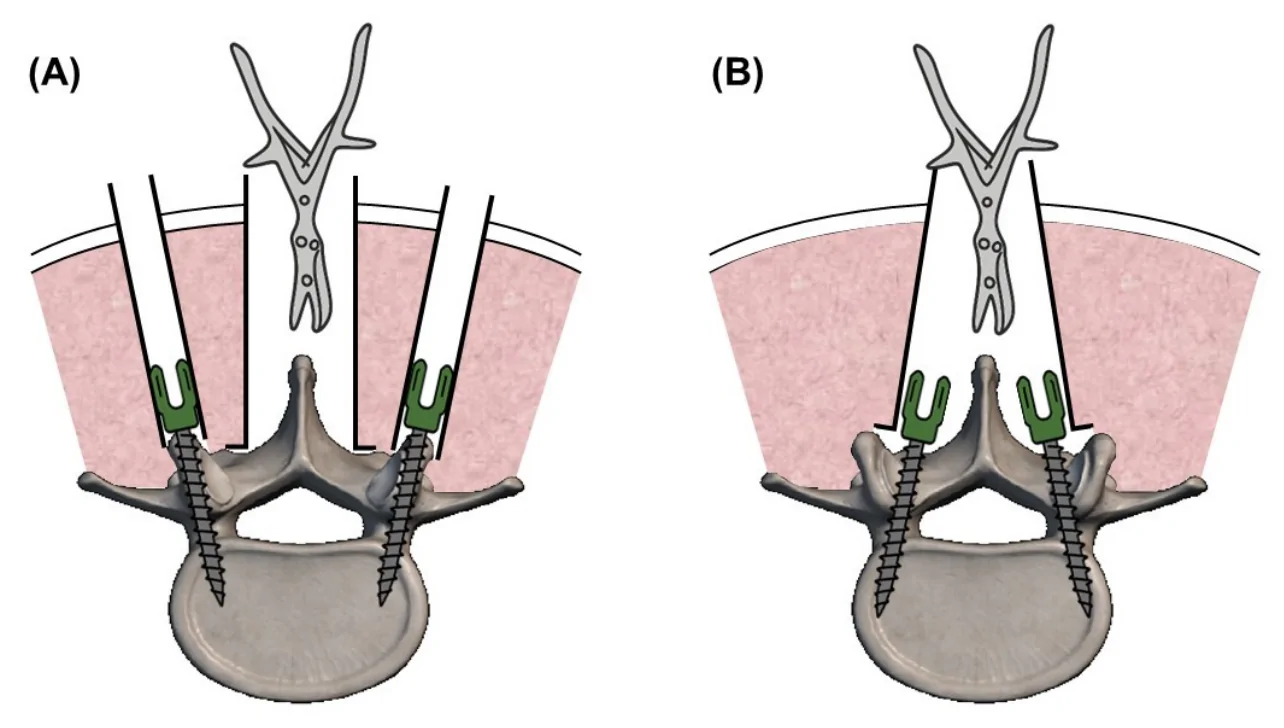
Posterior approach for screw insertion and bone graft harvesting. (**A**) In percutaneous pedicle screws (PPS) cases, bone graft harvesting is performed through a routine midline incision, whereas screw insertion requires four additional lateral skin incisions. (**B**) In cortical bone trajectory (CBT) cases, both screw insertion and bone graft harvesting are performed through a single midline incision without lateral skin incisions.

Percutaneous endoscopic transforaminal lumbar interbody fusion (PETLIF^®^), also referred to as full-endoscopic transforaminal approach lumbar interbody fusion (TF-LIF), is a minimally invasive fusion technique in which an interbody cage is inserted endoscopically through Kambin’s triangle [1,2]. This procedure typically uses percutaneous pedicle screws (PPS) for slip reduction and achieves indirect decompression by restoring disc height with an oval sleeve. Because direct decompression is not routinely required, the risk of postoperative hematoma is low, and drain placement is generally unnecessary. Previous studies have demonstrated the effectiveness of PETLIF for degenerative lumbar spondylolisthesis [3,4,5,6,7,8]. Achieving solid fusion, however, requires adequate autologous bone grafting [9,10]. Although iliac crest bone is traditionally used, donor-site pain and limited graft volume remain concerns [9,10,11,12,13]. To avoid these issues, our institution routinely harvests the spinous process through a small midline incision, providing sufficient graft volume without additional morbidity (Figure 1).

Although PETLIF relies primarily on indirect decompression, cases with severe facet degeneration or bony lateral recess stenosis may require direct decompression, which is also performed through the same midline incision. PPS insertion typically requires multiple lateral skin and fascial incisions, and previous studies have reported associations between soft tissue manipulation during PPS insertion or rod placement and postoperative pain [14,15]. Thus, further reduction of cumulative soft tissue handling remains desirable, even in PETLIF, which is inherently minimally invasive.

Cortical bone trajectory (CBT) screws were developed as a fixation technique for osteoporotic bone and have demonstrated excellent initial stability and high pullout strength [16,17,18,19,20]. Their medial-to-lateral trajectory allows screw insertion through a minimal midline incision with limited muscle dissection, thereby reducing paravertebral muscle damage [21,22,23]. In PETLIF, this approach enables screw insertion, direct decompression when indicated, and autologous bone harvesting to be performed through a single exposure. This consolidation of procedural steps may reduce overall tissue disruption. Recent meta-analytic evidence has demonstrated favorable perioperative profiles for percutaneous endoscopic transforaminal procedures compared with conventional approaches [24,25], supporting the minimally invasive principles underlying PETLIF. CBT may reduce muscle injury specifically within PETLIF because its midline cortical trajectory allows screw insertion with minimal paravertebral muscle dissection, whereas PPS requires lateral fascial splitting and muscle retraction. Previous comparative studies in other lumbar fusion techniques have reported lower postoperative CK elevation and reduced early postoperative pain with CBT, supporting its potential to decrease muscle trauma. CK was selected as the principal surrogate marker of muscle injury because it is widely used to quantify paravertebral muscle damage after lumbar fusion. When interpreting perioperative outcomes, it is important to distinguish technical or exposure-related advantages from demonstrated clinical benefits.

Despite the increasing use of CBT screws in minimally invasive lumbar fusion, no study has directly compared PPS and CBT fixation specifically in PETLIF, a technique with unique anatomical and procedural characteristics. Because PETLIF is designed as a minimally invasive procedure, understanding how screw trajectory influences perioperative invasiveness and early postoperative recovery is clinically meaningful. Therefore, the present study focused on short-term perioperative outcomes, including muscle injury and early postoperative pain, rather than long-term fusion or subsidence. The aim of this study was to compare short-term perioperative clinical outcomes between PPS and CBT screw insertion in PETLIF and to evaluate whether CBT screws may offer procedural advantages in this full-endoscopic fusion technique.

## 2. Materials and Methods

### 2.1. Study Design and Patients

This retrospective study analyzed 140 consecutive patients who underwent single-level PETLIF at L3/L4, L4/L5, or L5/S1 between July 2021 and June 2025. Exclusion criteria included trauma, infection, tumor, revision surgery, multilevel fusion, neuromuscular disorders, and preoperative CK elevation. Patients were categorized into two groups according to the screw insertion technique: the PPS group (N = 69) and the CBT group (N = 71). Direct decompression was performed when preoperative MRI or CT demonstrated severe facet degeneration, bony lateral recess stenosis, or foraminal stenosis not amenable to indirect decompression. The higher frequency of direct decompression in the CBT group reflected surgeon preference for performing decompression through the same midline exposure used for CBT screw insertion. To address its potential confounding effect, a subgroup analysis limited to patients without direct decompression was performed. The choice between PPS and CBT fixation was determined by surgeon preference based on patient anatomy, facet degeneration, bone quality, and the anticipated need for direct decompression. Both fixation techniques were used contemporaneously throughout the study period, and no chronological transition from PPS to CBT occurred. PETLIF procedures were performed by two experienced spine surgeons, each of whom routinely used both PPS and CBT fixation, minimizing surgeon specific and temporal bias. Baseline demographic and clinical variables included sex, age, BMI, ASA classification, and comorbidities such as hypertension, dyslipidemia, diabetes mellitus, chronic kidney disease, asthma, and history of malignancy. Lumbar spine bone mineral density was assessed using DEXA (Lunar iDXA; GE Healthcare, Madison, WI, USA), and t-scores were recorded as an index of baseline bone quality. Smoking status, use of immunosuppressive agents or systemic steroids, and antithrombotic medication use were also recorded to minimize confounding effects on perioperative CK and Hb changes. In addition, evaluated parameters included surgical characteristics, perioperative laboratory findings, and pre- and postoperative radiographic assessments. Patient-reported clinical outcome measures were also collected. All patients remained hospitalized for at least 7 days postoperatively, enabling complete collection of laboratory data at postoperative day (POD) 1, 3, and 7. Clinical questionnaires (VAS and JOA) were administered at each scheduled timepoint, and no missing data occurred. This study was approved by the Ethics Committee of our institution (IRB No. 022-0345), and informed consent was obtained from all participants at the time of surgery, where patients were informed that their clinical data might be used for research purposes. Because prospective consent had been obtained, additional consent for this retrospective analysis was not required.

### 2.2. Surgical Procedure

#### 2.2.1. Methods for Decompression and Cage Insertion

**Figure 2:**
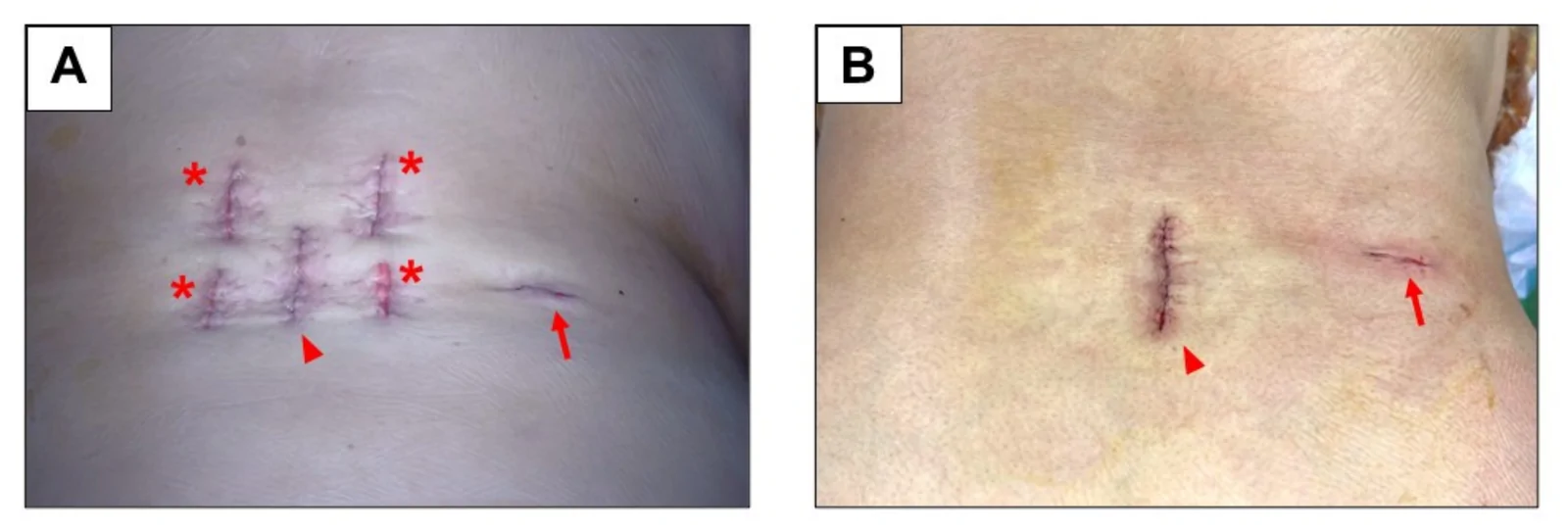
Posterior skin incisions in transforaminal lumbar interbody fusion cases. (**A**) In percutaneous pedicle screws (PPS) cases, a total of 6 skin incisions are required: one midline incision for bone harvesting and exposure, four lateral skin incisions for screw insertion, and one incision for endoscope insertion. (**B**) In cortical bone trajectory (CBT) cases, only two skin incisions are required: one midline incision for both screw insertion and bone harvesting, and one incision for endoscope insertion. Asterisks: incisions for PPS insertion. Red arrows: incisions for endoscope and cage insertion. Red triangles: midline incisions for harvesting bone graft (**A**) and CBT screw insertion and harvesting bone graft (**B**).

PETLIF was performed according to previously described techniques [2,8]. Under general anesthesia, patients were placed in the prone position on a radiolucent frame. Intraoperative neuromonitoring of the quadriceps and tibialis anterior muscles was performed using the NVM5 system (NuVasive, San Diego, CA, USA). Pedicle screws were inserted using either the PPS or CBT technique (Pisces; Japan Medical Dynamic Marketing, INC.: JMDM, Tokyo, Japan). Spinous processes were harvested through a midline incision for use as autologous graft material (Figure 2). Direct decompression was added in cases with preoperative imaging suggesting insufficient indirect decompression due to bony lateral recess stenosis.

Reduction of spondylolisthesis was achieved using screw-rod constructs, after which rods were inserted and set screws tightened. A skin incision was made approximately 7–8 cm lateral to the midline (distance from the midline), with an actual incision length of about 2–3 cm for the transforaminal endoscopic approach (Spine TIP Transforaminal Approach Kit; KARL STORZ, Tuttlingen, Germany). The endoscope was inserted at a 45° angle, which corresponds to the cage insertion angle. Foraminoplasty was performed using a high-speed drill to expand the Kambin’s triangle, which forms the cage entry point (Primado2; NAKANISHI, Tochigi, Japan) [26]. Next, the endoscope was advanced into the intervertebral disc, and after inserting a circular dilator, an oval dilator and sleeve were inserted to restore disc height. Under endoscopic visualization, disc material and cartilaginous endplates were removed. Autologous bone graft was packed through the sleeve, and an interbody cage (Vision Ti; JMDM, Tokyo, Japan) was inserted and compressed. The endoscopic transforaminal approach described above follows standardized steps, including foraminoplasty, disc preparation under continuous endoscopic visualization, and cage insertion through an oval working sleeve.

#### 2.2.2. PPS Technique

**Figure 3:**
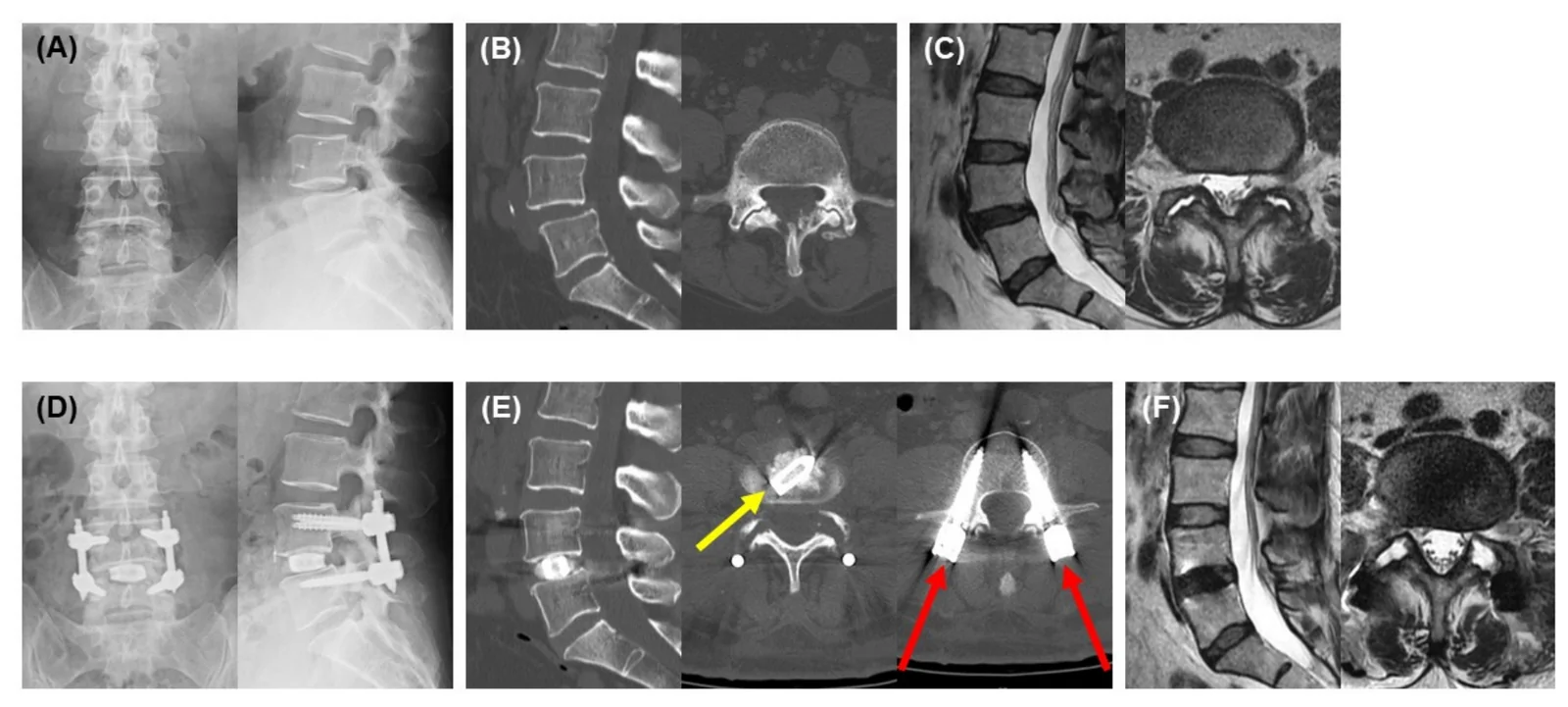
Representative case of transforaminal lumbar interbody fusion using percutaneous pedicle screws. (**A**) Preoperative X-ray images. (**B**) Preoperative computed tomography (CT) images. (**C**) Preoperative magnetic resonance (MR) images. (**D**) Postoperative X-ray images. (**E**) Postoperative CT images. Yellow arrow, cage insertion trajectory. Red arrows, screw insertion trajectory. (**F**) Postoperative MR images.

Four approximately 2 cm skin incisions were made over the planned screw entry points. After blunt dissection, the lateral aspect of the facet joint was palpated. Under fluoroscopic guidance, a needle with a sleeve was inserted, followed by guidewire placement, tapping, and screw insertion. A routine 4–5 cm midline incision was also made to harvest the spinous process and expose the interlaminar space. When direct decompression was required, it was performed through the same midline incision. Representative pre- and postoperative images are shown in Figure 3.

#### 2.2.3. CBT Screw Technique

**Figure 4:**
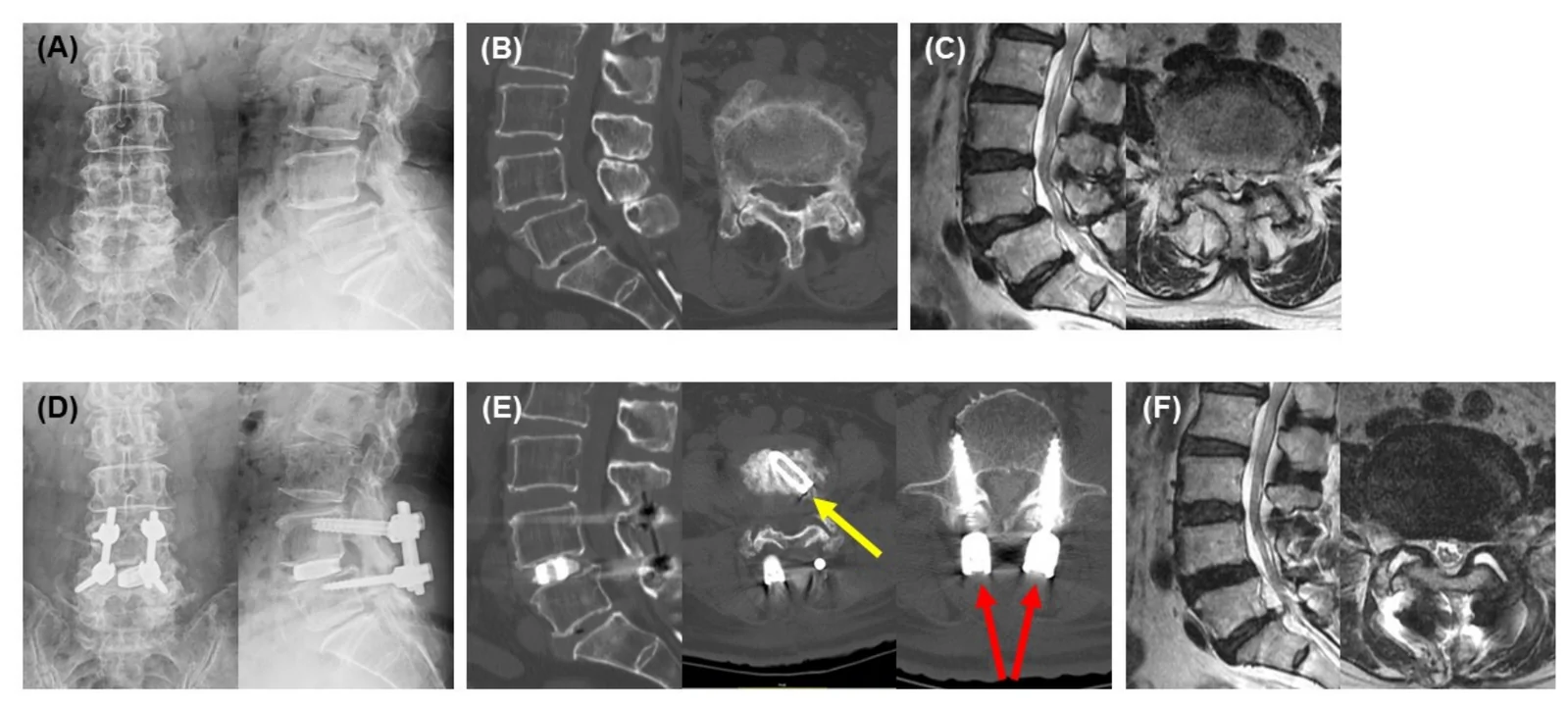
Representative case of transforaminal lumbar interbody fusion using cortical bone trajectory screws. (**A**) Preoperative X-ray images. (**B**) Preoperative computed tomography (CT) images. (**C**) Preoperative magnetic resonance (MR) images. (**D**) Postoperative X-ray images. (**E**) Postoperative CT images. Yellow arrow, cage insertion trajectory. Red arrows, screw insertion trajectory. (**F**) Postoperative MR images.

A single 4–5 cm midline incision was made, and the paravertebral muscles were dissected to expose the medial aspect of the facet joint. Under direct visualization and front and side fluoroscopy, a needle with a sleeve was inserted, followed by guidewire placement and screw insertion. When required, direct decompression and bone harvesting were performed through the same midline incision. Representative pre- and postoperative images are shown in Figure 4.

Screw trajectory selection (CBT or PPS) was based on surgeon preference considering patient anatomy, facet degeneration, bone quality, and anticipated need for direct decompression. Both techniques were used throughout the study period without a temporal transition, and annual case numbers were comparable. All procedures were performed by two experienced spine surgeons, each of whom routinely used both PPS and CBT. This non-randomized allocation introduces potential selection bias.

### 2.3. Assessment of Clinical Outcomes

Patient demographics and clinical outcomes were retrospectively evaluated. Surgical parameters included operative time, intraoperative blood loss, presence and extent of direct decompression, and perioperative complications. Operative time was defined as the duration from skin incision to skin closure, including all surgical steps such as endoscopic manipulation, screw insertion, and decompression. No intraoperative patient repositioning occurred in any case. Complications were defined as surgical site infection, dural tear, hematoma, or implant-related issues requiring reoperation.

Laboratory data, including hemoglobin (Hb) and creatine kinase (CK), were obtained preoperatively and on POD 1, 3, and 7. Percentage change in CK and Hb was calculated for each patient as (postoperative value − preoperative value)/preoperative value × 100, and reported as mean ± standard deviation. Radiographic evaluation included measurement of anterior slip percentage (% slip) and slip correction rate using preoperative and immediate postoperative lateral radiographs [27,28]. Clinical outcomes were assessed using the Japanese Orthopaedic Association (JOA) score (0–29; higher scores indicate better function) and visual analog scale (VAS) scores (0–10) for low back pain and leg pain, measured preoperatively and at POD 7 and POD 60.

### 2.4. Statistical Analysis

Data are expressed as mean ± standard deviation. Comparative statistical analyses were conducted between the PPS and CBT groups for patients’ background characteristics and imaging findings. Continuous variables were analyzed using the unpaired Student’s *t*-test. Binomial and categorical variables were evaluated using the chi-squared or Fisher’s exact test. Statistical significance was set at *p* < 0.05. Mean differences and 95% confidence intervals were calculated for intraoperative blood loss and Hb values. Normality was assessed using the Shapiro–Wilk test, and skewed variables were additionally evaluated using log-transformed values. Because CK values demonstrated a skewed distribution, between-group comparisons for CK and CK percentage change were performed using the Mann–Whitney U test (rank-based test) instead of the unpaired Student’s *t*-test. Generative AI tools (Microsoft Copilot, accessed in 2026) were used for language refinement during manuscript preparation.

## 3. Results

### 3.1. Demographics and Clinical Characteristics

A total of 140 patients were included in the analysis. The PPS group consisted of 69 patients (17 men and 52 women; mean age, 66.7 ± 10.6 years; range, 43–85 years), and the CBT group consisted of 71 patients (24 men and 47 women; mean age, 65.8 ± 11.2 years; range, 47–82 years). There were no significant differences in age or sex distribution. Baseline characteristics, including BMI, ASA classification, comorbidities, smoking status, and medication use, were comparable between groups (Table 1). These findings indicate that patient-related factors potentially affecting perioperative blood loss, CK elevation, or postoperative recovery were well balanced. Lumbar spine t-scores were also comparable between groups (PPS: −0.524 ± 1.151; CBT: −0.469 ± 1.367; *p* = 0.8236), indicating similar baseline bone quality (Table 1). Operated levels were L3/L4 (PPS: 13; CBT: 10), L4/L5 (PPS: 54; CBT: 59), and L5/S1 (PPS: 2; CBT: 2), with no significant intergroup differences (Table 1). Degenerative spondylolisthesis was the most common diagnosis in both groups (PPS: 66; CBT: 66), followed by degenerative scoliosis (PPS: 2; CBT: 3) and foraminal stenosis (PPS: 1; CBT: 2). The mean length of hospital stay was 8.3 ± 0.5 days in both groups, and all patients remained hospitalized for at least 7 days, resulting in complete POD 1, 3, and 7 laboratory and questionnaire data without missing values (Table 1).

### 3.2. Surgical Outcomes

Operative time was slightly shorter in the CBT group (124.8 ± 25.3 min) than in the PPS group (131.7 ± 23.7 min), although the difference was not statistically significant (*p* = 0.1398, Table 2). Intraoperative blood loss was significantly higher in the CBT group (121.2 ± 71.5 mL) compared with the PPS group (79.4 ± 61.1 mL) (*p* = 0.0005), with a mean difference of 41.8 mL (95% CI 19.5–64.0) (Table 2). Direct decompression was performed significantly more often in the CBT group (38 cases) than in the PPS group (21 cases) (*p* = 0.0056, Table 2). Among these, one-level decompression was performed in 19 PPS and 27 CBT cases, whereas two level decompression was performed in two PPS and 11 CBT cases. In the subgroup analysis of patients without direct decompression, blood loss was 81.1 ± 33.8 mL in the CBT group and 69.8 ± 34.9 mL in the PPS group (mean difference 11.3 mL, 95% CI −0.2 to 22.7), with no significant difference between groups (*p* = 0.1566, Table 2). In contrast, in the decompression group, blood loss was 156.1 ± 74.2 mL in the CBT group and 102.4 ± 53.3 mL in the PPS group (*p* = 0.0057), with a mean difference of 53.7 mL (95% CI 31.2–76.1). Perioperative complications were rare, with only one case of surgical site infection in the PPS group and none in the CBT group (Table 2).

### 3.3. Perioperative Laboratory Data

Laboratory findings showed no significant differences in Hb levels between groups at any time point (preoperative, POD 1, POD 3, POD 7) (Table 3). Similarly, the percentage change in Hb from baseline did not differ significantly (mean differences and 95% confidence intervals are presented in Table 3). In contrast, CK levels on POD 1 were significantly higher in the PPS group (753.0 ± 276.7 U/L) than in the CBT group (539.3 ± 322.4 U/L) (Mann–Whitney U test, *p* = 0.0136) (Table 3). Although CK levels on POD 3 and POD 7 did not differ significantly, the percentage change in CK from baseline remained significantly higher in the PPS group at POD 1 (*p* = 0.0087) and POD 3 (*p* = 0.0493) (Table 3). In the subgroup analysis limited to patients without direct decompression, postoperative CK elevation remained significantly lower in the CBT group compared with the PPS group (Table S1).

### 3.4. Radiographical Outcomes

Radiographically, the percentage of slip improved from 18.9 ± 5.4% to 8.4 ± 5.5% in the PPS group and from 19.5 ± 5.2% to 7.5 ± 5.3% in the CBT group, with no significant differences between groups (Table 4). Slip correction rates were similar (PPS: 58.1 ± 25.0%; CBT: 61.7 ± 23.5%), although the CBT group showed a non-significant trend toward greater correction (Table 4).

### 3.5. Clinical Outcomes

Clinical outcomes showed no significant differences in JOA scores between groups at any time point (Table 4). Leg pain VAS scores were also comparable. Low back pain VAS scores did not differ preoperatively or at POD 60; however, on POD 7, the CBT group demonstrated significantly lower scores than the PPS group (2.3 ± 1.9 vs. 3.2 ± 2.2; *p* = 0.0187), with a mean difference of −0.9 (95% CI −1.6 to −0.2) (Table 4). In the subgroup analysis limited to patients without direct decompression, early postoperative low back pain on POD 7 remained significantly lower in the CBT group compared with the PPS group (Table S2).

## 4. Discussion

This study compared short-term perioperative outcomes between PPS and CBT screw fixation in PETLIF, a full-endoscopic transforaminal fusion technique designed to minimize soft tissue disruption. The two groups demonstrated comparable baseline characteristics, operated levels, radiographic slip correction, and functional recovery based on JOA scores, indicating that patient-related and anatomical factors were well balanced. These similarities support an association between screw insertion technique and the observed differences in perioperative muscle injury and early postoperative pain; however, residual confounding related to the retrospective design and differences in concomitant direct decompression cannot be excluded.

A key finding of this study was the significantly lower CK elevation in the CBT group, particularly on POD 1, with consistently smaller percentage changes from baseline across all postoperative time points. CK elevation is widely used as an indicator of paravertebral muscle injury, and previous studies have reported increased CK release following PPS insertion due to lateral fascial dissection and muscle retraction [14,15]. In contrast, CBT screws can be inserted through a single midline incision with limited muscle dissection [21,22,23]. The present results support these biomechanical and anatomical advantages, demonstrating that CBT fixation reduces short-term muscle injury even within the minimally invasive PETLIF framework. However, CK is a transient and systemic marker affected by multiple non-specific factors. To definitively conclude that CBT reduces muscle injury, the evaluation of lumbar MR images (e.g., postoperative lumbar MRI measuring multifidus muscle cross-sectional area or fatty infiltration) should be investigated in future studies. Postoperative MRI was available; however, the scans were acquired for routine clinical evaluation and lacked standardized parameters necessary for quantitative assessment of multifidus cross-sectional area or T2 signal change. As a result, MRI-based muscle injury analysis could not be performed retrospectively, and dedicated imaging protocols will be required in future studies. VAS and JOA assessments were obtained by investigators blinded to the fixation technique.

Early postoperative low back pain was also significantly lower in the CBT group on POD 7. This finding is consistent with the reduced muscle injury observed in the CBT group and aligns with previous reports linking paravertebral muscle preservation to improved early postoperative comfort [21,22,23]. The reduction in early postoperative low back pain observed in the CBT group may relate to decreased paravertebral muscle disruption, consistent with recent comprehensive evaluations of discogenic low back pain mechanisms [29]. Although pain scores at POD 60 were comparable, the early reduction in low back pain may facilitate faster mobilization and smoother recovery during the immediate postoperative period. Direct decompression was performed more frequently in the CBT group, raising the possibility of confounding. To clarify its influence, we conducted a subgroup analysis limited to patients without direct decompression. In this subgroup, CBT fixation still showed lower postoperative CK elevation and reduced early postoperative low back pain compared with PPS fixation. These findings indicate that the advantages of CBT fixation were not solely attributable to decompression-related tissue handling and support an independent association between screw trajectory and perioperative muscle injury. Given that decompression differed between groups and no adjusted analysis was performed, the association between screw trajectory and reduced muscle injury should be interpreted as suggestive rather than conclusive. These subgroup results are presented in Supplemental Tables S1 and S2, confirming that CK and VAS differences persisted even when restricting the analysis to patients without direct decompression. Although the difference in low back pain VAS at POD 7 reached statistical significance, the absolute magnitude (0.9 points) is smaller than commonly reported minimal clinically important difference (MCID) thresholds for postoperative lumbar spine pain (approximately 1.5–2.0 points) [30]. Therefore, this early pain reduction should be interpreted cautiously, particularly given that longer-term pain and JOA outcomes were comparable between groups. Because postoperative low back pain in this study reflects new pain arising from surgical tissue handling rather than improvement from preoperative baseline, MCID-based responder analysis was not applicable. The absolute difference at POD 7 (0.9 points), although statistically significant, was interpreted cautiously given that it falls below commonly reported MCID thresholds. These findings should also be interpreted within the broader context of lumbar muscle function and rehabilitation. Systematic reviews have highlighted the importance of muscle endurance, fatigue assessment, and structured rehabilitation in patients with spinal disorders, suggesting that postoperative muscle preservation may influence early functional recovery [31,32]. However, because direct decompression differed significantly between groups, this represents a major source of confounding, and the main analysis did not include adjusted or multivariable modeling to account for this imbalance.

Although intraoperative blood loss was higher in the CBT group, a subgroup analysis showed no significant difference between the two groups in the non-decompression group; however, in the decompression group, blood loss was significantly higher in the CBT group. Given that the CBT group had a larger number of cases in which decompression was performed and that decompression was frequently performed involving multiple levels, it was suggested that the increase in blood loss in the CBT group was attributable to the performance of multilevel decompression rather than the trajectory/incision. Additionally, it is important that postoperative Hb changes were comparable between groups, suggesting that the increased intraoperative blood loss did not translate into clinically meaningful anemia or delayed recovery.

Radiographic outcomes, including slip correction and correction rate, were similar between groups. These findings indicate that both PPS and CBT fixation provide adequate mechanical stability for slip reduction in PETLIF. Because CBT screws rely on cortical purchase, baseline bone quality is relevant to their mechanical behavior. Lumbar spine t-scores were comparable between groups, indicating that both fixation constructs were evaluated under similar bone density conditions.

One procedural characteristic of CBT fixation in PETLIF is that screw insertion, direct decompression when indicated, and autologous bone harvesting can be performed through a single midline incision. This configuration reduces the need for multiple lateral skin incisions required for PPS insertion and represents a theoretical simplification of the surgical sequence. Although operative time did not differ significantly between groups, this single-incision approach may reduce cumulative soft tissue handling; however, it should not be interpreted as a demonstrated improvement in surgical efficiency. Advances in minimally invasive spine surgery continue to emphasize reduced tissue disruption and improved surgical precision. Robotic-assisted systems such as the Da Vinci platform illustrate these broader technological trends across surgical disciplines [33,34]. In lumbar surgery, however, endoscopic transforaminal approaches remain the primary minimally invasive pathway, enabling targeted decompression and interbody preparation through limited soft tissue exposure.

To strengthen the interpretation of group differences, mean differences and 95% confidence intervals were added for intraoperative blood loss, Hb, and CK values. These additions provide clearer information regarding the magnitude and precision of the observed effects. Although repeated measures or mixed effects modeling may offer further analytical advantages, the retrospective design and available dataset were not suited for multivariable longitudinal analysis; therefore, postoperative changes in Hb, CK, JOA, and VAS were interpreted descriptively, and multiple comparisons were considered exploratory. Normality was assessed using the Shapiro–Wilk test, and skewed variables such as CK and blood loss were additionally evaluated using log-transformed values.

This study has several limitations. Its retrospective design introduces potential selection bias, and direct decompression was performed more frequently in the CBT group, reflecting surgeon preference rather than random allocation. Although CK and Hb were measured repeatedly within the same patients, a repeated-measures or mixed-effects model was not applied. Each time point was analyzed independently, which may not fully account for within-patient correlations. Additionally, multiple comparisons were performed without formal adjustment, and the possibility of type I error inflation cannot be excluded. In particular, the significant POD 7 low back pain result may not remain significant under standard correction methods. Long-term outcomes such as fusion rates and implant-related complications were not evaluated. Future prospective studies with standardized decompression criteria and long-term radiological follow-up are warranted. Recent predictive modeling studies have highlighted biomechanical factors associated with cage subsidence in lumbar interbody fusion procedures [35]. Although our study did not evaluate mid- to long-term cage behavior, these findings underscore the importance of future longitudinal PETLIF studies assessing fusion quality and cage stability. Furthermore, the retrospective and non-randomized design may introduce temporal or surgeon-related confounding, and the imbalance in direct decompression represents an additional source of bias. Adjusted analyses were not performed, and follow-up was relatively short, without assessment of long-term fusion, subsidence, screw loosening, or implant-related complications. CK served as an indirect surrogate of muscle injury rather than a direct imaging-based evaluation, and postoperative rehabilitation or analgesic protocols may have influenced early pain outcomes. Finally, although POD 7 low back pain VAS differed significantly between groups, the magnitude did not exceed commonly reported MCID thresholds.

In summary, CBT fixation in PETLIF resulted in reduced short-term muscle injury and lower early postoperative low back pain compared with PPS fixation, while maintaining comparable radiographic and functional outcomes. The ability to perform screw insertion, decompression, and bone harvesting through a single midline incision represents a procedural characteristic of CBT fixation in full-endoscopic lumbar fusion.

## 5. Conclusions

Within the limitations of this retrospective comparative study, CBT fixation in PETLIF was associated with lower early postoperative CK elevation and lower low back pain scores at POD 7 compared with PPS fixation, while radiographic correction and short-term functional outcomes were comparable. The use of a single midline exposure may offer procedural advantages; however, prospective studies with standardized decompression criteria, adjustment for relevant confounders, and long-term follow-up are required to confirm these findings.

## Figures and Tables

**Table 1:** Characteristics of the patients in the percutaneous pedicle screw group and the cortical bone trajectory group.

	Percutaneous Pedicle Screw Group (N = 69)	Cortical Bone Trajectory Group (N = 71)	*p*-Value
Number of patients (male/female)	69 (17/52)	71 (24/47)	0.2335
Age (range)	66.7 ± 10.6 (43–85)	65.8 ± 11.2 (47–82)	0.6721
BMI (kg/m^2^)	24.5 ± 3.7	24.2 ± 3.4	0.6422
ASA class (I/II/III)	5/61/3	6/63/2	0.8630
Comorbidities (+/−)	64/5	65/6	0.7912
Hypertension	44	40	0.3696
Dyslipidemia	41	42	0.9745
Diabetes mellitus	9	7	0.5538
Chronic kidney disease	9	7	0.5538
Asthma	4	5	0.7639
History of malignancy	5	8	0.4124
Lumbar spine t-score	−0.524 ± 1.151	−0.469 ± 1.367	0.8236
Length of hospital stay (days)	8.3 ± 0.5	8.3 ± 0.5	0.8236
Current smoker	14	16	0.7462
Immunosuppressive/steroid use	1	2	0.5764
Antithrombotic medication use	11	7	0.2824
Surgical levels	L3/4: 13	L3/4: 10	0.7467
	L4/5: 54	L4/5: 59	
	L5/S1: 2	L5/S1: 2	
Diagnosis			0.7769
Degenerative spondylolisthesis	66	66	
Degenerative scoliosis	2	3	
Foraminal stenosis	1	2	

All data represent mean ± SD.

**Table 2:** Surgical outcomes in the percutaneous pedicle screw group and the cortical bone trajectory group. Mean differences (CBT − PPS) and 95% confidence intervals (CI) were calculated for estimated blood loss.

	Percutaneous Pedicle Screw Group (N = 69)	Cortical Bone Trajectory Group (N = 71)	Mean Difference (CBT − PPS)	95% CI	*p*-Value
Operative time (min)	131.7 ± 23.7	124.8 ± 25.3			0.1398
Estimated blood loss (mL)					
total	79.4 ± 61.1	121.2 ± 71.5	41.8	19.5~64.0	0.0005
non-decompression	69.8 ± 34.9	81.1 ± 33.8	11.3	−0.2~22.7	0.1566
decompression	102.4 ± 53.3	156.1 ± 74.2	53.7	31.2~76.1	0.0057
Direct decompression					0.0056
none	48	33			
one level	19	27			
two levels	2	11			
Complications					
Infection	1	0			0.3087

All data represent mean ± SD.

**Table 3:** Hemoglobin (Hb) and creatine kinase (CK) values during the perioperative period in the percutaneous pedicle screw group and the cortical bone trajectory group. Mean differences and 95% CI were calculated for Hb values, whereas CK values and CK percentage change were compared using the Mann–Whitney U test.

	Percutaneous Pedicle Screw Group (N = 69)	Cortical Bone Trajectory Group (N = 71)	Mean Difference (CBT − PPS)	95% CI	*p*-Value
Hb (g/dL)					
Preoperative	13.2 ± 1.4	13.5 ± 1.5	0.21	−0.27~0.69	0.4431
POD 1	11.7 ± 1.2	11.6 ± 1.8	−0.02	−0.52~0.50	0.9555
POD 1 − Δ Pre (%)	−11.7 ± 6.9	−13.6 ± 8.6	−1.8	−4.4~0.8	0.2274
POD 3	11.3 ± 1.2	11.5 ± 1.8	0.16	−0.35~0.67	0.5922
POD 3 − Δ Pre (%)	−14.2 ± 6.5	−14.6 ± 8.6	0.4	−2.9~2.2	0.7968
POD 7	11.4 ± 1.3	11.7 ± 2.3	−0.01	−0.30~0.89	0.3985
POD 7 − Δ Pre (%)	−13.8 ± 6.1	−13.2 ± 11.4	0.6	−2.5~3.6	0.7430
CK (U/L)					
Preoperative	95.8 ± 59.8	110.7 ± 46.0			0.2538
POD 1	753.0 ± 276.7	539.3 ± 322.4			0.0136
POD 1 − Δ Pre (%)	826.1 ± 505.7	489.2 ± 534.6			0.0087
POD 3	584.3 ± 256.7	462.6 ± 270.4			0.1168
POD 3 − Δ Pre (%)	638.1 ± 468.8	383.4 ± 333.9			0.0493
POD 7	124.0 ± 52.8	114.9 ± 44.0			0.4141
POD 7 − Δ Pre (%)	45.9 ± 73.5	13.7 ± 51.3			0.1136

Actual measured values at preoperative, postoperative day 1, 3, and 7, and the percentage change from preoperative levels. All data represent mean ± SD.

**Table 4:** Radiographical and clinical outcomes in the percutaneous pedicle screw group and the cortical bone trajectory group. Mean differences and 95% CI were calculated for JOA score, Leg pain VAS values.

	Percutaneous Pedicle Screw Group (N = 69)	Cortical Bone Trajectory Group (N = 71)	Mean Difference (CBT − PPS)	95% CI	*p*-Value
% slip					
Preoperative (%)	18.9 ± 5.4	19.5 ± 5.2			0.8167
Postoperative (%)	8.4 ± 5.5	7.5 ± 5.3			0.7161
Correction rate (%)	58.1 ± 25.0	61.7 ± 23.5			0.7248
JOA score					
Preoperative	15.7 ± 4.6	14.3 ± 5.0	−1.3	−2.9~0.3	0.1468
POD 7	22.0 ± 3.7	21.9 ± 3.8	−0.1	−1.3~1.2	0.9313
POD 60	25.4 ± 3.2	25.4 ± 3.5	0.1	−1.1~1.1	0.9711
Leg pain VAS					
Preoperative	6.5 ± 2.4	7.2 ± 2.1	0.7	−0.1~1.4	0.1242
POD 7	2.3 ± 2.0	1.6 ± 2.1	−0.7	−1.4~0.1	0.0674
POD 60	1.6 ± 1.8	1.4 ± 2.2	−0.2	−0.9~0.4	0.5951
Low back pain VAS					
Preoperative	5.7 ± 2.9	6.1 ± 2.8	0.4	−0.6~1.3	0.4738
POD 7	3.2 ± 2.2	2.3 ± 1.9	−0.9	−1.6~−0.2	0.0187
POD 60	2.1 ± 1.9	2.3 ± 2.5	0.3	−0.5~1.0	0.5703

All data represent mean ± SD.

## Data Availability

The data presented in this study are available from the corresponding author upon request. The data are not publicly available due to patient privacy and institutional regulations.

## Supplementary Materials

The following supporting information can be downloaded at: https://www.mdpi.com/article/10.3390/jcm15187174/s1, Table S1: Creatine kinase (CK) values during the perioperative period in the percutaneous pedicle screw group and the cortical bone trajectory group without decompression. Actual measured values at preoperative, postoperative day 1, day 3, and day 7, and the percentage change from preoperative levels. CK values and CK percentage change were compared using the Mann–Whitney U test. All data represent mean ± SD; Table S2: Clinical outcomes in the percutaneous pedicle screw and the cortical bone trajectory group without decompression. All data represent mean ± SD.

## Abbreviations

The following abbreviations are used in this manuscript:

PETLIF	Percutaneous endoscopic transforaminal lumbar interbody fusion
PPS	Percutaneous pedicle screws
CBT	Cortical bone trajectory
Hb	hemoglobin
CK	Creatine kinase
JOA	Japanese Orthopaedic Association
VAS	Visual analog scale
TF-LIF	Full-endoscopic transforaminal approach lumbar interbody fusion
POD	Postoperative days
