# Supplementary material for: Comparison of Cortical Bone Trajectory and Percutaneous Pedicle Screw Fixation in PETLIF: Short-Term Perioperative Muscle Injury and Pain Outcomes
Source: J Clin Med. 2026 Sep 15;15(18):7174. doi: 10.3390/jcm15187174 (PMC13607272; doi:10.3390/jcm15187174)
Supplement: Supplementary file 1 [file jcm-15-07174-s001.zip › Table S1.pdf]

Table S1. Creatine kinase (CK) values during the perioperative period in the percutaneous pedicle screw group and the cortical bone trajectory group without decompression. Actual measured values at preoperative, postoperative day 1, day 3, and day 7, and the percentage change from preoperative levels. CK values and CK percentage change were compared using the Mann–Whitney U test. All data represent mean ± SD.

	Percutaneous pedicle screw group (N = 48)	Cortical bone trajectory group (N = 33)	p-value
CK (U/L)			
Preoperative	100.81 ± 62.44	121.18 ± 40.25	0.1902
POD 1	763.69 ± 236.83	609.18 ± 347.22	0.1300
POD 1 - Δ Pre (%)	810.1 ± 481.7	429.1 ± 308.8	0.0065
POD 3	592.31 ± 242.38	534.36 ± 293.56	0.5221
POD 3 - Δ Pre (%)	624.9 ± 469.2	387.0 ± 320.9	0.0793
POD 7	129.50 ± 55.50	121.68 ± 44.24	0.6357
POD 7 - Δ Pre (%)	46.5 ± 78.8	4.5 ± 38.1	0.0409

CK; creatine kinase, Pre; preoperative, POD; postoperative day
