# Supplementary material for: Comparison of Cortical Bone Trajectory and Percutaneous Pedicle Screw Fixation in PETLIF: Short-Term Perioperative Muscle Injury and Pain Outcomes
Source: J Clin Med. 2026 Sep 15;15(18):7174. doi: 10.3390/jcm15187174 (PMC13607272; doi:10.3390/jcm15187174)
Supplement: Supplementary file 1 [file jcm-15-07174-s001.zip › Table S2.pdf]

Table S2. Clinical outcomes in the percutaneous pedicle screw and the cortical bone trajectory group without decompression. All data represent mean ± SD.

	Percutaneous pedicle screw group (N = 48)	Cortical bone trajectory group (N = 33)	p-value
JOA score			
Preoperative	16.1 ± 5.0	14.3 ± 5.2	0.1822
POD 7	22.1 ± 3.7	22.3 ± 3.5	0.8618
POD 60	26.1 ± 3.1	25.8 ± 3.2	0.6974
Leg pain VAS			
Preoperative	6.6 ± 2.1	7.3 ± 2.4	0.2440
POD 7	2.3 ± 2.1	1.2 ± 1.8	0.0377
POD 60	1.5 ± 1.8	1.0 ± 1.4	0.2486
Low back pain VAS			
Preoperative	5.2 ± 2.8	6.0 ± 2.9	0.3209
POD 7	3.4 ± 1.9	2.2 ± 1.9	0.0176
POD 60	1.9 ± 1.8	2.1 ± 2.2	0.7242

VAS; Visual analogue scale, POD; Postoperative day
